# SIRT1 reduction is associated with sex-specific dysregulation of renal lipid metabolism and stress responses in offspring by maternal high-fat diet

**DOI:** 10.1038/s41598-017-08694-4

**Published:** 2017-08-21

**Authors:** Long The Nguyen, Hui Chen, Carol Pollock, Sonia Saad

**Affiliations:** 1 Renal medicine, Kolling Institute, Royal North Shore Hospital, University of Sydney, Sydney, New South Wales Australia; 2 School of Life Sciences, Faculty of Science, University of Technology Sydney, Sydney, New South Wales Australia

## Abstract

Rodent models of maternal obesity have been associated with kidney damage and dysfunction in offspring. However, the underlying mechanisms are yet to be elucidated. In this study, female rats were fed a high-fat diet (HFD) for 6 weeks prior to mating, throughout gestation and lactation; both male and female offspring were examined at weaning. Our results demonstrate that renal lipid deposition was increased in male offspring only, which is associated with reduced protein expression of Sirtuin (SIRT) 1, an essential regulator of lipid metabolism and stress response. Other components in its signalling network including phosphorylated 5′-AMP-activated protein kinase (pAMPKα), Forkhead box FOXO3a and Peroxisome proliferator-activated receptor (PPAR)γ coactivator 1-alpha (PGC-1α) were also downregulated. By contrast, in female offspring, renal fat/lipid distribution was unchanged in coupling with normal SIRT1 regulation. Specific autophagy and antioxidant markers were suppressed in both sexes. On the other hand, fibronectin and Collagen type IV protein expression was significantly higher in the offspring born HFD-fed dams, particularly in the males. Collectively, these findings suggest that maternal HFD consumption can induce sex-specific changes in offspring kidney lipid metabolism and stress responses at early ages, which may underpin the risk of kidney diseases later in life.

## Introduction

Chronic kidney disease (CKD) is a global health crisis, characterised by a gradual loss of kidney function and ultimately requiring renal replacement therapy. Up to 90% loss of kidney function can occur before any symptoms are perceived^1^. There is increasing recognition that a predisposition to CKD is evident in children and young adults, although overt renal failure may not manifest till older age is reached^2^. Such phenomenon has been attributed in part to “fetal programming”, a term for fetal metabolic and epigenetic alterations induced by an abnormal intrauterine environment^3–5^. Maternal obesity during pregnancy is one of the factors that can induce fetal programming, leading to increased body weight and adiposity in the offspring^6, 7^, and pathophysiological changes in the kidney in adulthood^8^. Oxidative stress and inflammation have been identified as two main factors contributing to such outcomes^9^. Nevertheless, the changes are often detected at later stages when other permanent damages may have already occurred^9^. Therefore, it is important to identify earlier targets for a preventative therapy.

Lipid metabolism and autophagy are among the earliest possible mechanisms that can be affected by maternal obesity considering their direct relevance to energy intake and dietary composition. Indeed, perirenal fat and intrarenal lipid accumulation can be used as predicting factors for renal fibrosis and CKD^10, 11^. On the other hand, autophagy is negatively regulated by nutrition availability, which functions to dispose of and recycle damaged and dysfunctional cellular components that otherwise accumulate and interfere with cellular homeostasis and activities. Prolonged deficiency in autophagy for example in obesity and aging increase renal susceptibility to glomerular and tubulointerstitial pathology^12, 13^. Apart from the two pathways, antioxidant shortage is a direct cause of oxidative imbalance that can result in oxidative damage and cellular dysfunction in the offspring.

Sirtuins (SIRTs) are a group of d NAD^+^-dependent deacetylases and mono-ADP-ribosyl transferases. In mammals, seven members of the family (SIRT1–7) have been identified, among which SIRT1 is the most extensively studied, having pivotal roles in multiple processes especially stress responses and glucose/fat metabolism^14^. Its expression and activities are known to be promoted during nutrient deprivation^15^ and suppressed when nutrient is in excess, such as in obese individuals^16^. With regard to the kidney, activation of the SIRT1 signalling pathway has been shown to reduce renal lipotoxicity^17^, improve renal autophagy^18^ and antioxidant defence^19^, thereby attenuate kidney diseases in obese and diabetic animals.

In models of maternal obesity, it has been shown that SIRT1 expression is reduced in the fetus^20^ and specifically in the offspring liver^21^, suggesting a potential role of SIRT1 as a mediator of the programming effects by maternal obesity^22^. Nevertheless, the regulation of renal SIRT1 signalling in the offspring has yet to be demonstrated. In this study, we examine the hypothesis that maternal obesity induces renal lipid metabolic disorders and autophagy deficiency in the offspring and SIRT1 signalling plays a central role in this mechanism. As the risk of CKD differs between sexes^23^ and so does the fetal programming of kidney disorders^24–27^, we also examined whether these mechanisms are regulated in a sex-specific manner.

## Results

### Maternal HFD consumption increased body weight, fat deposition, plasma glycerides and kidney weight in both male and female offspring

At weaning, HFD-fed dams had significantly higher body weight, retroperitoneal fat mass and liver weight compared to chow-fed dams (P < 0.05, Table 1). Both male and female offspring of the HFD-fed dams showed significantly greater body weight than those born to chow-fed dams (P < 0.05, Table 2). Moreover, female offspring were significantly smaller than males (P < 0.05, sex effect). Mesenteric, retroperitoneal and perigonadal fat mass in both MHF male and female offspring was significantly higher in the offspring from HFD-fed dams (P < 0.05). The difference remained significant when adjusted for body weight (P < 0.01) except for male offspring’s mesenteric fat. Similar to the body weight, the overall percentage of retroperitoneal fat in female offspring was significantly smaller than in the males (P < 0.05, sex effect). The net liver weight was significantly increased by MHF only in female offspring. The sex effect for liver weight was significant (P < 0.05, sex effect), as well as the interaction between sex and maternal diet (P < 0.05, interaction). Kidney weight was also significantly increased in the offspring (P < 0.05 in male and P < 0.01 in female respectively). Overall, the females have lighter kidneys than the males. Consistent with the increased body weight and adiposity, plasma triglyceride levels were also increased in both sexes in the offspring (P < 0.05 and P < 0.01 respectively), whereas the levels of non-esterified fatty acid were unchanged.

**Table 1:** Body weight and organ mass of dams fed with Chow or HFD.

	Chow-fed dam (n = 5)	HFD dam (n = 6)
Body weight at weaning (g)	320 ± 35.09	362 ± 25.41*
Retroperitoneal fat (g)	5.62 ± 2.61	11.9 ± 3.31**
Retroperitoneal fat %	1.71 ± 0.60	3.31 ± 0.93**
Perigonadal fat (g)	5.92 ± 1.23	6.23 ± 0.83
Perigonadal fat %	1.92 ± 0.31	1.73 ± 0.28
Mesenteric fat (g)	4.44 ± 0.41	5.50 ± 1.30
Mesenteric fat %	1.39 ± 0.12	1.52 ± 0.34
Liver (g)	10.53 ± 1.34	15.13 ± 1.54**
Liver %	3.29 ± 0.19	4.19 ± 0.38**

Results are expressed as means ± SD. Data were analysed by student t-test. *P < 0.05, **P < 0.01.

**Table 2:** Body weight and organ mass of male and female offspring at weaning.

	Male		Female		2-way ANOVA P-values
	MC (n = 12)	MHF (n = 13)	MC (n = 12)	MHF (n = 13)	
BW (g)	49.6 ± 8.3	60.3 ± 10.8*	40.9 ± 3.5†	57.7 ± 7.2**	a, b
Mesenteric fat (g)	0.40 ± 0.12	0.59 ± 0.18*	0.28 ± 0.09	0.56 ± 0.12**	a
Mesenteric fat (%)	0.82 ± 0.13	0.94 ± 0.17	0.69 ± 0.20	0.97 ± 0.10**	a
Retroperitoneal fat (g)	0.08 ± 0.02	0.33 ± 0.17**	0.04 ± 0.02	0.28 ± 0.15**	a
Retroperitoneal fat (%)	0.15 ± 0.03	0.52 ± 0.21**	0.10 ± 0.04	0.48 ± 0.22**	a, b
Perigonadal fat (g)	0.08 ± 0.03	0.26 ± 0.12**	0.06 ± 0.03	0.30 ± 0.11**	a
Perigonadal fat (%)	0.15 ± 0.04	0.42 ± 0.13**	0.15 ± 0.06	0.50 ± 0.14**	a
Liver (g)	1.67 ± 0.16	1.90 ± 0.47	1.57 ± 0.26	2.47 ± 0.44**	a, b, c
Liver (%)	3.43 ± 0.41	3.21 ± 0.76	3.82 ± 0.35	4.29 ± 0.54	b, c
Kidney (g)	0.27 ± 0.05	0.33 ± 0.06*	0.23 ± 0.03	0.30 ± 0.06**	a, b
Kidney (%)	0.55 ± 0.03	0.54 ± 0.04	0.56 ± 0.04	0.59 ± 0.05	a
Plasma TG (g/L)	0.31 ± 0.08	0.47 ± 0.13*	0.30 ± 0.11	0.47 ± 0.07**	a
Plasma NEFA (mM)	0.50 ± 0.10	0.62 ± 0.25	0.58 ± 0.18	0.64 ± 0.11	ns

Results are expressed as means ± SD. The data were analysed using two-way ANOVA with Bonferroni post hoc test. MC (offspring of dams fed chow); MHF (offspring of dams fed high-fat diet). *P < 0.05, **P < 0.01 (vs MC offspring); ^†^P < 0.05 (vs male offspring), ^a^(P < 0.05, Maternal diet effect), ^b^(P < 0.5, Sex effect), ^c^(P < 0.05, interaction effect). BW (Body weight), BGL (Blood glucose level).

### Maternal HFD consumption increased perirenal fat deposition and renal triglyceride concentration in male but not female offspring

**Figure 1:**
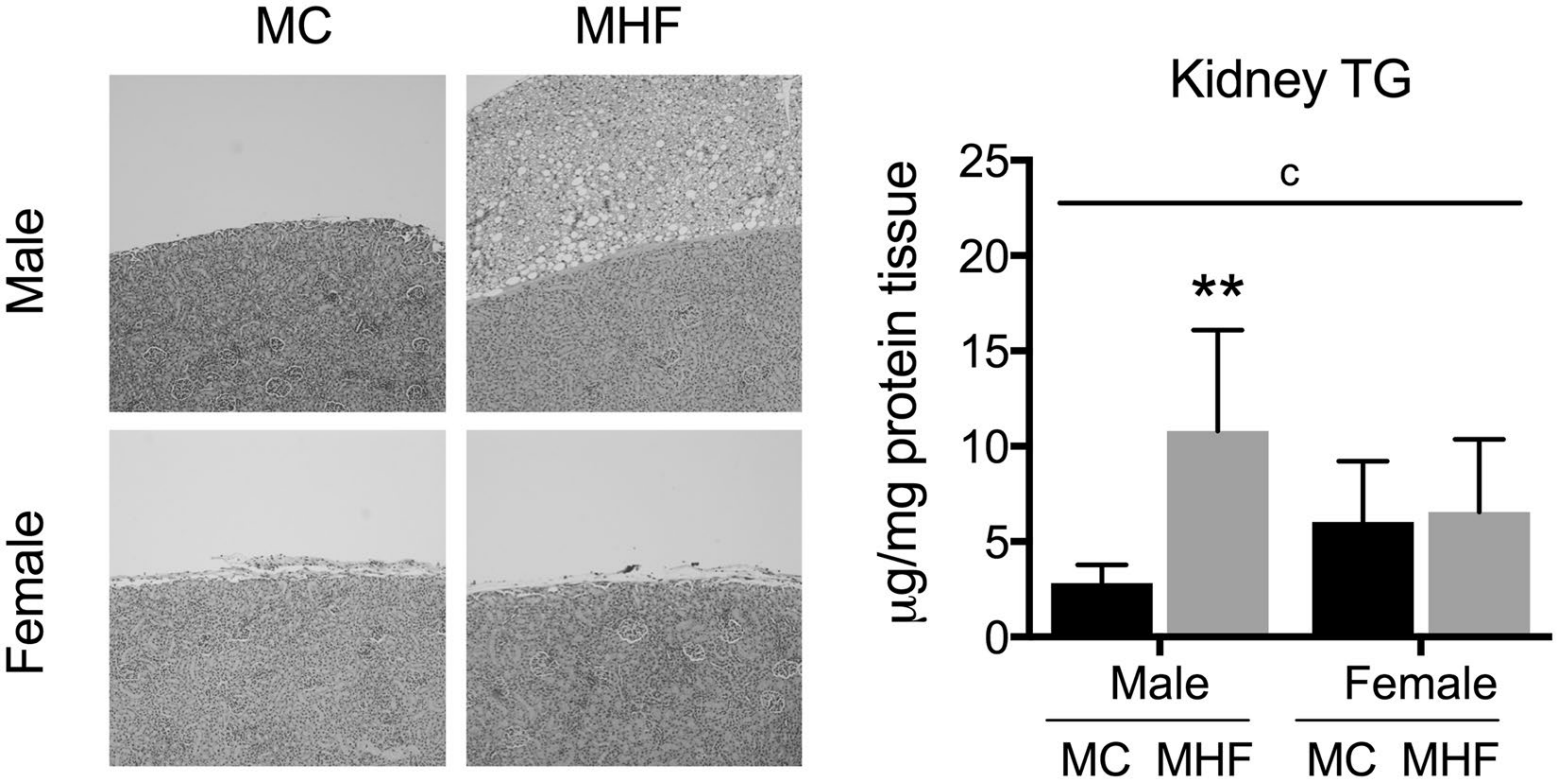
Maternal high-fat diet increases perirenal fat deposition and triglyceride concentration in male but not female offspring. MC (offspring of dams fed chow); MHF (offspring of dams fed high-fat diet). Results are expressed as mean ± SD. Data were analysed by two-way ANOVA followed by Bonferroni post hoc test. (**a**) (P < 0.05, Maternal diet effect), (**b**) (P < 0.5, Sex effect), (**c**) (P < 0.05, interaction effect), **P < 0.01 (vs MC offspring). n = 5.

Male offspring from HFD-fed dams demonstrated increased perirenal fat deposition (Fig. 1), which was unchanged in female offspring. Similarly, kidney levels of triglycerides in male offspring were significantly higher when born to dams fed a HFD (P < 0.01), whereas in female offspring, no difference was observed between the groups. The sex-specific effect is reflected by a significant interaction (P < 0.05) between the two factors: sex and maternal diet.

### The effects of maternal HFD consumption on lipid metabolism regulators in offspring kidney

As kidney triglyceride levels were increased in the male offspring only, we examined the mRNA expression of potential regulator(s) involved in lipid metabolism. As shown in Table 3, only mRNA expression of SIRT1 and PGC-1α was significantly downregulated in the male offspring due to maternal HFD consumption (P < 0.01 and P < 0.05 respectively). In female offspring, no differences at the transcriptional level were detected. The sex-specific regulation of SIRT1 mRNA is reflected by a significant interaction (P < 0.05) between the two factors of sex and maternal diet.

**Table 3:** mRNA expression of lipid metabolism markers in offspring kidney.

	Male		Female		2-way ANOVA P-values
	MC (n = 6)	MHF (n = 6)	MC (n = 6)	MHF (n = 6)	
SIRT1	0.885 ± 0.177	0.649 ± 0.052**	0.752 ± 0.123	0.794 ± 0.148	c
PGC-1a	0.997 ± 0.209	0.670 ± 0.059*	0.941 ± 0.264	0.905 ± 0.207	a
PPARa	1.177 ± 0.498	0.762 ± 0.209	0.619 ± 0.1188^†^	0.6 ± 0.249	b
PPARg1	1.128 ± 0.922	1.006 ± 0.613	2.261 ± 1.921	2.187 ± 0.885	b
SREBP1c	1.007 ± 0.293	1.045 ± 0.072	0.987 ± 0.414	0.892 ± 0.251	ns
FAS	0.822 ± 0.176	0.856 ± 0.170	0.956 ± 0.371	0.93 ± 0.064	ns
FABP3	0.936 ± 0.128	1.146 ± 0.449	1.426 ± 0.630	1.414 ± 0.193	b

MC (offspring of dams fed chow); MHF (offspring of dams fed high-fat diet). SIRT1 **(**Sirtuin1); PPARγ (Peroxisome proliferator-activated receptor gamma); PPARα (Peroxisome proliferator-activated receptor alpha); PGC-1α (PPARγ coactivator 1-alpha); FAS (Fatty acid synthase); SREBP-1c (Sterol regulatory element-binding protein); Fatty acid binding protein (FABP) 3. Results are expressed as means ± SD. Data were analysed using two-way ANOVA with Bonferroni post hoc test. *P < 0.05, **P < 0.01 (vs MC offspring), ^†^P < 0.05 (vs male offspring). ^a^(P < 0.05, Maternal diet effect), ^b^(P < 0.5, Sex effect), ^c^(P < 0.05, interaction effect).

Together with SIRT1 and PGC-1α, PPARα and PPARγ are also essential regulators of lipid metabolism. In this study, we showed that the mRNA expression of PPARα in the offspring kidney were generally lower in females (P < 0.05, sex effect, Table 3). By a sharp contrast, the levels of PPARγ were significantly higher in the females (P < 0.05, sex effect). The effect of maternal HFD consumption on the transcription of the two genes was minor except a slight tendency of downregulation of PPARα in the MHF males (Table 3).

Similar to PPARγ, the overall mRNA expression of FABP3 was also significantly higher in the females (P < 0.05, sex effect) and unchanged by maternal HFD consumption. No difference in the renal expression of other regulators of the lipogenesis pathway including SREBP1c and FAS were detected.

### Maternal HFD consumption regulates renal SIRT1 signalling network in the offspring in a sex-specific manner

**Figure 2:**
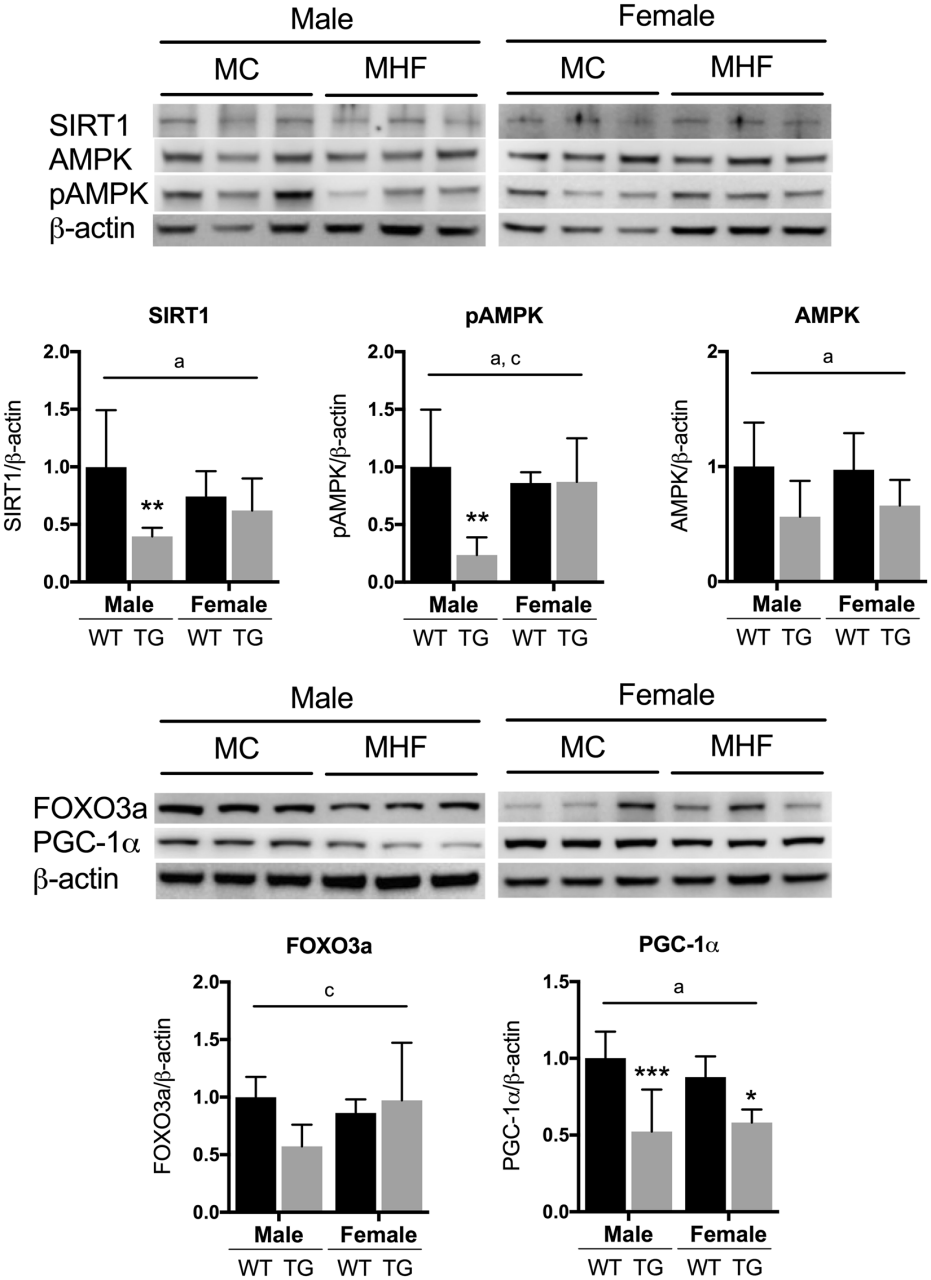
Protein levels of markers of SIRT1 signalling network in male and female offspring kidney. MC (offspring of dams fed chow); MHF (offspring of dams fed high-fat diet). SIRT1 (Sirtuin), PGC-1α (Peroxisome proliferator-activated receptor gamma coactivator 1-alpha), AMPKα (5′ AMP-activated protein kinase alpha), pAMPKα (Phosphorylated AMPKα) and FOXO3a (Forkhead box O3a). Results are expressed as mean ± SD. Data were analysed by two-way ANOVA followed by Bonferroni post hoc test. (**a**) (P < 0.05, Maternal diet effect), (**b**) (P < 0.5, Sex effect), (**c**) (P < 0.05, interaction effect), *P < 0.05, **P < 0.01 (vs MC offspring). n = 6.

To confirm our observations, the protein expression of SIRT1 and other markers of its signalling pathway were analysed. Our results demonstrated that SIRT1 protein level was indeed reduced in the kidney of MHF male offspring (P < 0.05, Fig. 2), whereas in the females, no change was detected. The level of total AMPK, a positive regulator of the SIRT1 signalling network, was substantially reduced by approximately 50% (P = 0.052) in the MHF males. More importantly, the level of its active phosphorylated form pAMPK was also significantly suppressed correspondingly (P < 0.05). In the female offspring, the renal levels of AMPK and pAMPK were both unaffected. The sex-maternal diet interactions were near-significant (P = 0.07) and significant (P < 0.05) for SIRT1 and pAMPK respectively.

Similarly, the expression of FOXO3a, a downstream regulator of SIRT1, was also regulated in such interactive fashion (P < 0.05) with a near-significant reduction in MHF male offspring (P = 0.053). Interestingly, despite the unchanged mRNA expression in MHF females, the protein levels of PGC-1α were significantly reduced by maternal HFD consumption in both sexes (P < 0.001 and P < 0.05 respectively, Fig. 2).

**Figure 3:**
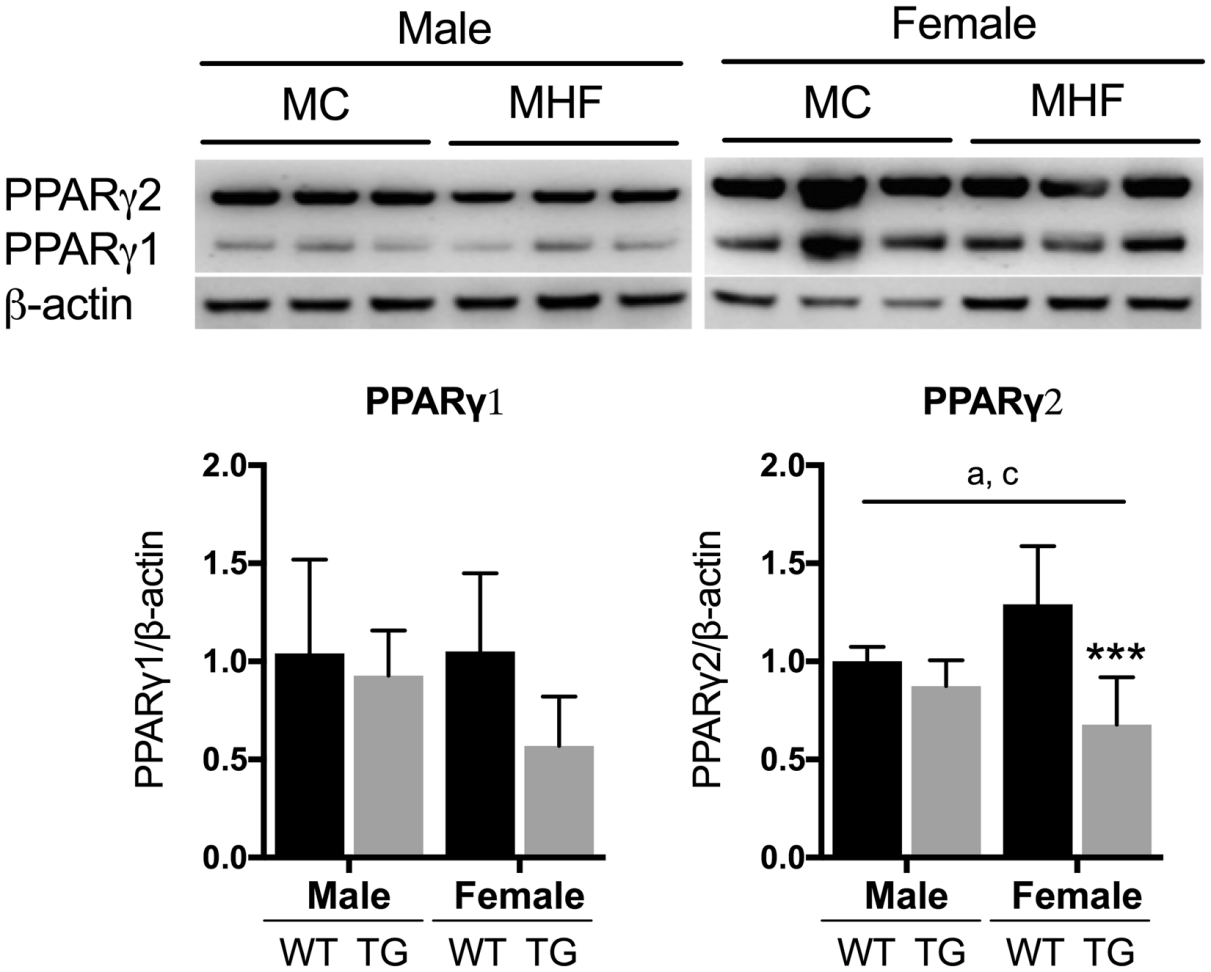
Protein levels of renal Peroxisome proliferator-activated receptor gamma isoform 1 and 2 in male and female offspring. MC (offspring of dams fed chow); MHF (offspring of dams fed high-fat diet). Results are expressed as mean ± SD. Data were analysed by two-way ANOVA followed by Bonferroni post hoc test. (**a**) (P < 0.05, Maternal diet effect), (**c**) (P < 0.05, interaction effect), **P < 0.01 (vs MC offspring). n = 6.

SIRT1 is known as a suppressor of PPARγ transcription^28^. However, in this study, the expression of PPARγ seems to be independent of SIRT1 signalling. The protein expression of both PPARγ isoforms 1 and 2 were substantially reduced (P = 0.059 and P < 0.01 respectively, Fig. 3) in MHF female offspring kidney. However, in the male offspring, no change was detected. The sex-specific effect is reflected by a significant interaction between the two factors of sex and maternal diet for the regulation of PPARγ2 (P < 0.05).

### Maternal HFD consumption downregulates renal autophagy markers

**Figure 4:**
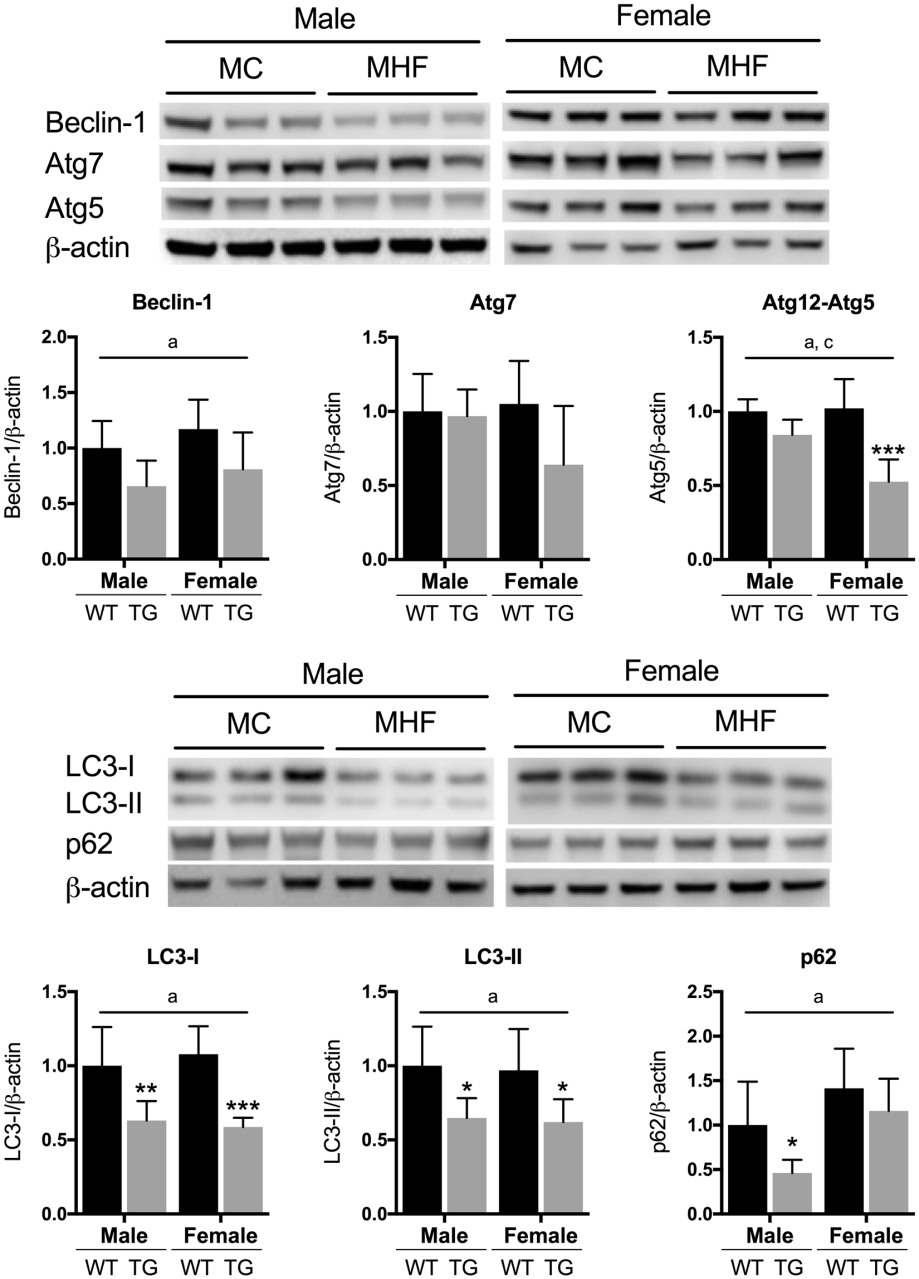
Regulation of renal autophagy markers in male and female offspring. MC (offspring of dams fed chow); MHF (offspring of dams fed high-fat diet). Atg (Autophagy-related protein); LC3-I (Microtubule-associated proteins 1A/1B light chain 3), LC3-II (lipidated LC3); p62 (Sequestome 1). Results are expressed as mean ± SD. Data were analysed by two-way ANOVA followed by Bonferroni post hoc test. (**a**) (P < 0.05, Maternal diet effect), (**b**) (P < 0.5, Sex effect), (**c**) (P < 0.05, interaction effect), *P < 0.05, ***P < 0.001 (vs MC offspring). n = 6.

Autophagy can be regulated by SIRT1 in multiple ways: either through the action of FOXO3a to upregulate the transcription of autophagy-related genes^29^, or by direct deacetylation of Atg proteins thereby increasing their activity^30^. Our results indicate that maternal HFD consumption has significant impacts on the renal expression of essential autophagy markers including Beclin-1, Atg12-Atg5 complex, LC3-I, LC3-II and p62 (P < 0.05, maternal diet effect, Fig. 4). Notably, while Beclin-1, LC3-I and II were suppressed to the same levels in both sexes, Atg12-Atg5 and p62 were regulated in a sex-specific fashion with significant reductions of the former in the female (P < 0.001) and the latter in the male offspring (P < 0.05) respectively. There was also a trend of reduction in the expression of Atg7 only in the females (Fig. 4).

### Maternal HFD consumption reduces renal levels of antioxidant marker GPx-1

**Figure 5:**
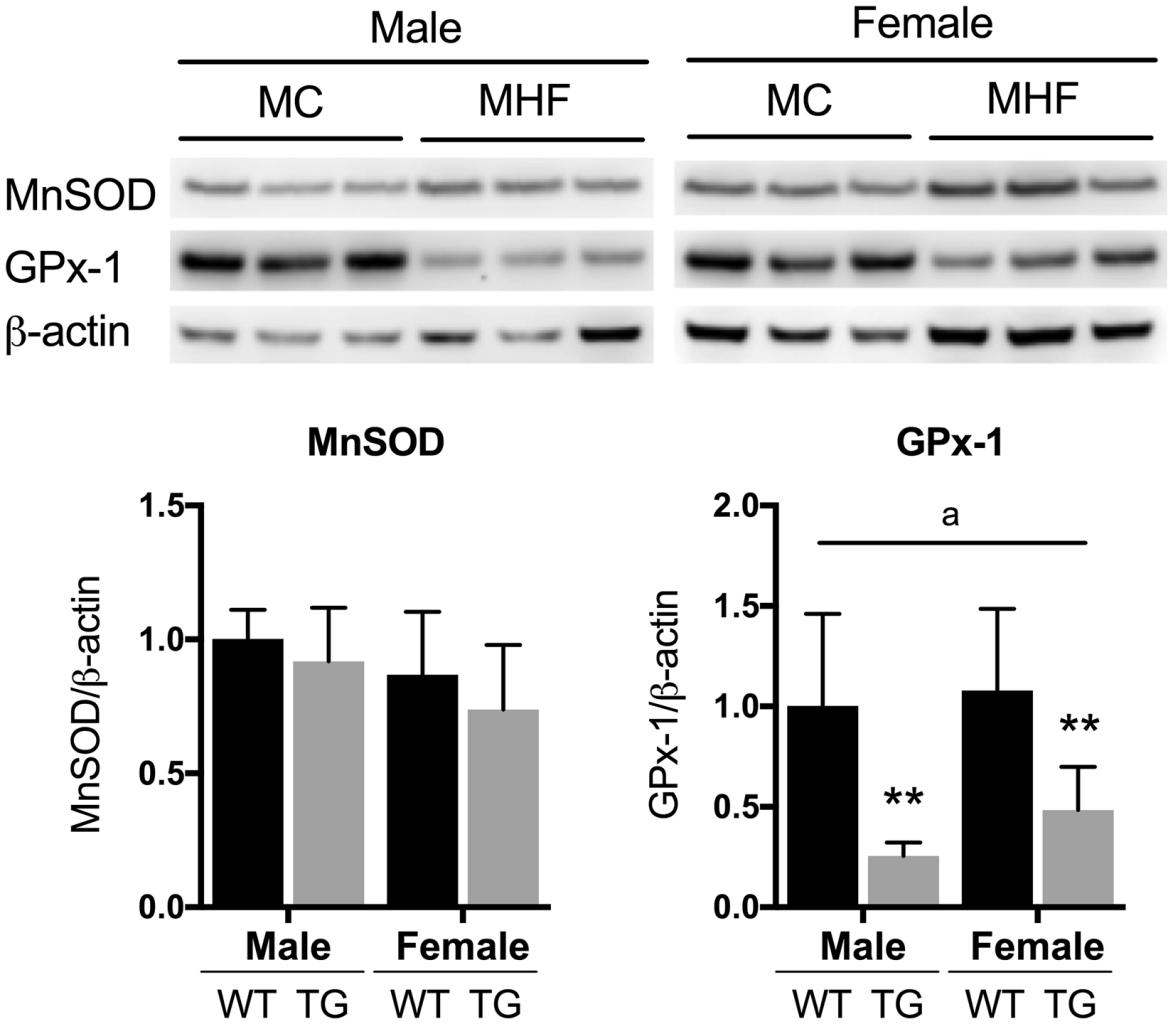
Regulation of renal antioxidant markers in male and female offspring. MC (offspring of dams fed chow); MHF (offspring of dams fed high-fat diet). MnSOD (Manganese superoxide dismutase); GPx1 (Glutathione Peroxidase 1). Results are expressed as mean ± SD. Data were analysed by two-way ANOVA followed by Bonferroni post hoc test. ^a^(P < 0.05, Maternal diet effect), **P < 0.01 (vs MC offspring). n = 6.

SIRT1 signalling has been associated with cellular antioxidant defence. MnSOD and GPx-1 were examined as representative markers of antioxidant defence, each of which is involved in one of the two-step reaction to convert reactive oxygen species into water and oxygen (O^−^_2_ → H_2_O_2_ → H_2_O + O_2_). As shown in Fig. 5, while the renal levels of MnSOD were not affected by maternal HFD consumption, GPx-1 proteins were significantly and consistently reduced in the offspring in both sexes (P < 0.01).

### The effects of maternal HFD consumption on renal markers of inflammation and extracellular matrix

**Figure 6:**
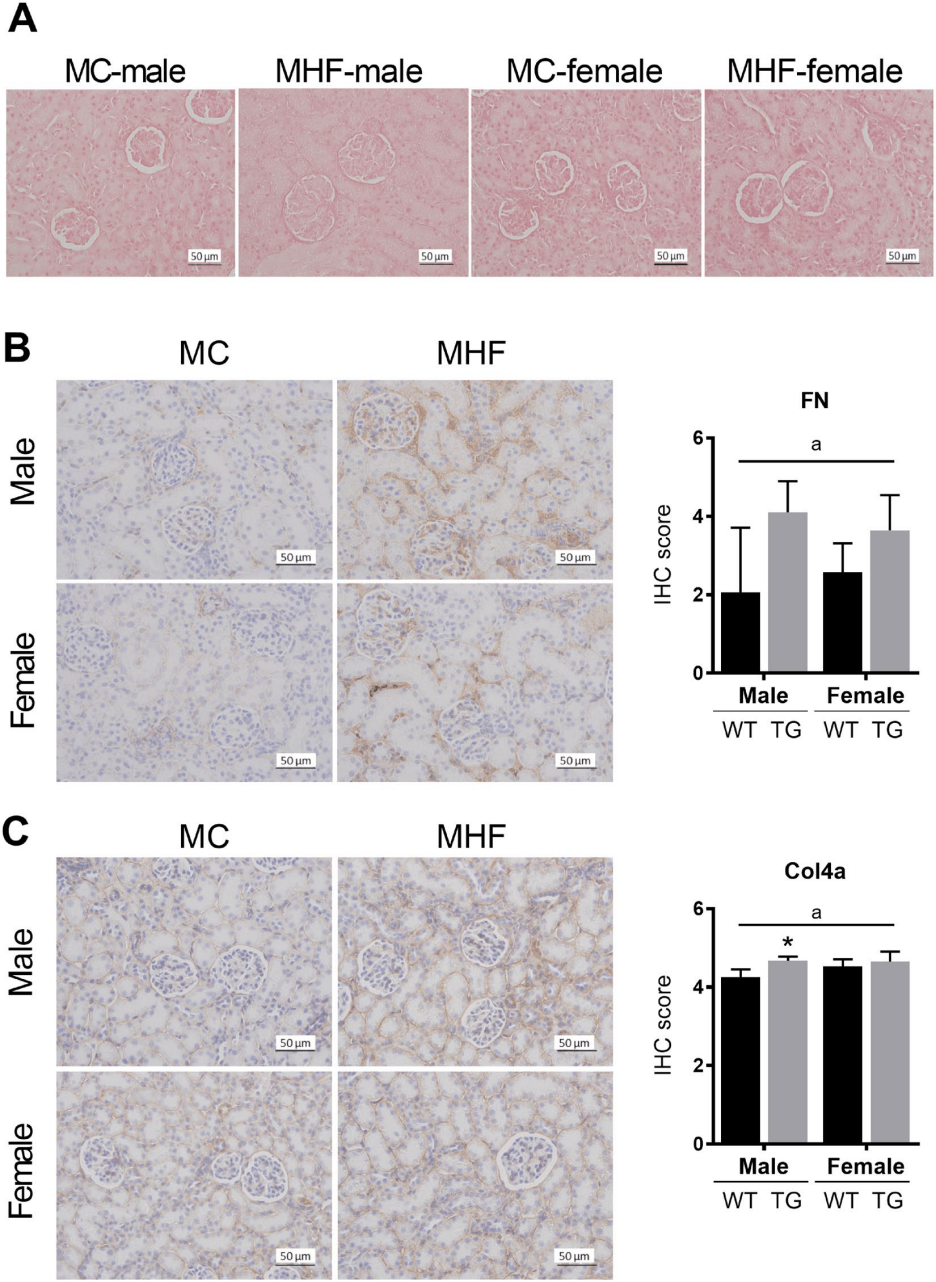
Renal histological staining in male and female offspring. (**A**) Picro Sirius Red staining of collagen type I and III. (**B**) Fibronectin immunohistochemistry. (**C**) Collagen type IV immunohistochemistry. MC (offspring of dams fed chow); MHF (offspring of dams fed high-fat diet). FN (Fibronectin); Col4 (Collagen type IV). Results are expressed as mean ± SD. Data were analysed by two-way ANOVA followed by Bonferroni post hoc test. ^a^(P < 0.05, Maternal diet effect), *P < 0.05 (vs MC offspring). n = 5.

Obesity is often associated with a low-grade inflammation^31^. Particularly in the kidney, it is associated with the elevation of TGFβ1 as well as structural modifications including glomerulosclerosis and interstitial fibrosis^32, 33^. In this study, TGFβ1 mRNA expression in the male offspring kidney was not affected by maternal HFD consumption. Higher levels of TGFβ1 were detected in the females (P < 0.05, sex effect, Table 4). By contrast, the renal expression of TGFβ receptor 1 was reduced in the kidney of MHF offspring (P < 0.05, maternal diet effect) and not different between the two sexes. Similarly, Col3A mRNA expression was gender-dependent (P < 0.05, sex effect), whereas Col1A was reduced by maternal HFD consumption (P < 0.05, maternal diet effect). Fibronectin mRNA expression showed a trend of increase by maternal HFD consumption (Table 4). In addition, immunohistochemistry staining also indicated an overall elevation of fibronectin protein in the offspring born to HFD-fed dams (Fig. 6B, P < 0.05, maternal diet effect). The renal protein level of Col4 was also significantly increased in MHF offspring (Fig. 6C, P < 0.05, maternal diet effect), especially in the males (P < 0.05).

**Table 4:** mRNA expression of inflammatory and fibrotic markers in offspring kidney.

	Male		Female		2-way ANOVA P-values
	MC (n = 6)	MHF (n = 6)	MC (n = 6)	MHF (n = 6)	
TGFβ1	1.039 ± 0.300	0.981 ± 0.123	1.496 ± 0.410	1.671 ± 0.622	b
TGFβR1	1.035 ± 0.265	0.723 ± 0.118	0.976 ± 0.252	0.876 ± 0.208	a
Col1A	1.015 ± 0.196	0.894 ± 0.284	1.071 ± 0.201	0.790 ± 0.170	a
Col3A	1.025 ± 0.252	1.032 ± 0.275	1.740 ± 0.386^†^	1.366 ± 0.576	b
FN	1.047 ± 0.345	1.268 ± 0.649	1.081 ± 0.164	1.208 ± 0.180	ns

MC (offspring of dams fed chow); MHF (offspring of dams fed high-fat diet). TGFβ1 (Transforming growth factor beta), TGFβR1 (TGFβ receptor 1), Col (Collagen), FN (Fibronectin). Results are expressed as means ± SD. Data were analysed using two-way ANOVA with Bonferroni post hoc test. *P < 0.05, **P < 0.01 (vs MC offspring), ^†^P < 0.05 (vs male offspring). ^a^(P < 0.05, Maternal diet effect), ^b^(P < 0.5, Sex effect), ^c^(P < 0.05, interaction effect).

## Discussion

Maternal high-fat diet consumption and obesity have been associated with kidney damage and dysfunction in the offspring in adulthood^8, 9^, which are likely the result of accumulative cellular/molecular events occurred during early developmental periods. In this study, we attempted to clarify the underlying mechanisms. We demonstrate that maternal overnutrition is associated with increased lipid deposition and the suppression of multiple stress responses including autophagy, antioxidant defence and inflammation in the offspring kidney with a significant relevance to the SIRT1 signalling network. In addition, the study also suggests that many of these early changes are sex-dependent.

Consistent with previous studies^6–8, 34^, maternal HFD consumption preconception, during gestation and laction significantly increased ofsspring body weight, fat deposition, plasma triglyceride level and kidney weight at weaning, suggesting increased caloric intake and cell proliferation. Male offspring showed higher body weight and fat mass compared to females, which is also in line with previous studies in rodents^26^ and human^35^, suggesting a higher tendency of males for obesity development. In addition, the fact that maternal HFD consumption significantly increased the liver weight of female but not male offsrping may imply a higher capacity of female liver for growth and perhaps also lipid storage and processing, which in turn may reduce lipid spillover to other tissues such as kidney.

Indeed, the levels of perirenal fat as well as intrarenal lipid content were higher only in MHF males but not females, suggesting the effects of maternal HFD consumption on offspring kidney are also sex-specific. Sex-dependent differences in fetal programming of nephropathy are well-established in maternal undernutritional conditions^24, 26, 27^ and to a lesser extend maternal overnutrition, for instance, a high-protein diet^25^. Increased intrarenal lipid content has been associated with glomerulopathy and tubulointerstitial sclerosis in obese patients^10^, while perirenal lipid deposition has also been suggested as an independent predictor of CKD^11^. Such early changes in lipid metabolism are likely to induce lipotoxicity and predispose to kidney dysfunction later in life, especially when a second insult to the kidney occurs, such as the development of diabetes or obesity in the offspring^5, 8^.

SIRT1 and its downstream factor PGC-1α are two stimuli of fatty acid oxidation (52, 53). As a consequence of maternal HFD consumption, renal mRNA and protein levels of SIRT1 and PGC-1α levels were reduced specifically in the male offspring, which may explain the male-specific elevation of renal fat. Together with SIRT1 and PGC-1α, the protein levels of phosphorylated AMPK was also downregulated. AMPK can indirectly activate SIRT1 by increasing the NAD^+^/NADH ratio through the upregulation of Nicotinamide phosphoribosyltransferase^36^. Conversely, SIRT1 is also required for AMPK activity^37^. As renal AMPK has been shown to be reduced in obesity and diabetes^17, 38^, its reduced expression and activation due to maternal obesity is consistent with the downregulation of SIRT1 signalling and increased lipid deposition in the male offspring kidney. Both SIRT1 and AMPK are activators of FOXO3a, while FOXO3a can also promote SIRT1 transcription by interfering with the suppressor p53 on the SIRT1 promoter^39^. As such, a positive feedback loop is formed within the SIRT1 signalling network.

In MHF female offspring, there was no change in renal expression of SIRT1, pAMPK or FOXO3a. Alternatively, the protein level of PPARγ2 was significantly reduced. As PGC-1α is a co-activator of PPARγ, its reduced level in MHF females supports downregulated PPARγ signalling. Although PPARγ is well-known as an indispensable controller of adipocyte differentiation and insulin sensitivity^40^, its role in the kidney is not clearly understood. It has been shown that PPARγ^+/−^ mice showed resistance to obesity-associated kidney lipid accumulation and injuries^41^. The reduction of renal PPARγ2 reduction in MHF female offspring may reflect an adaptive response to the increased fatty acid influx due to maternal HFD consumption. The regulation of PPAR signalling by maternal overnutrition has been previously reported in a transcriptome study^42^.

In this study, the protein levels of multiple autophagy markers were significantly reduced in the offspring kidney, suggesting suppression of autophagic processes. Hence, accumulation of misfolded proteins and dysfunctional organelles such as malfunctioning mitochondria is likely to occur^43^. Although autophagy was suppressed by maternal HFD consumption in both sexes, there were sex-dependent differences in the regulation of individual markers. The fact that the reduction of Atg12-Atg5 complex only occurred in the MHF females suggests that autophagosome elongation was likely to be impaired. On the other hand, p62 expression was only affected in the MHF males, indicating autophagosome degradation was interfered specifically in MHF male offspring. Prolonged deficiency in autophagy can increase renal susceptibility to glomerular and tubulointerstitial pathology^12, 13^.

Antioxidant defence systems have a close relationship with autophagy as both are essentially involved in the adaptive responses to oxidative stress^44, 45^. In this study, together with autophagy, renal antioxidant defence was also suppressed, reflected in the marked reduction in GPx-1 levels. The fact that the expression of MnSOD was unchanged indicates that the impairment of antioxidant defence was only partial and may not necessarily lead to oxidative stress at such young neonatal age. However, there is a certainly higher risk for oxidative stress and inflammation to occur in the offspring kidney if the defects is prolonged. Indeed, our group has recently demonstrated that maternal obesity can exacerbate kidney oxidative stress, inflammation and fibrosis induced by type 1 diabetes in the offspring^5^.

It has been shown that the SIRT1/AMPK/FOXO3a axis positively regulates autophagy^43, 46, 47^ and antioxidant defence^48, 49^, thereby preventing oxidative damage, particularly in kidney tubular cells^50, 51^. As such, the reduction of these markers in male MHF offspring kidney is likely to underline the deficiency of kidney autophagy and antioxidant defence markers. In female offspring, as the renal levels of SIRT1, pAMPK and FOXO3a were normal, the alterations in autophagy and antioxidant defence are likely to be mediated through a different pathway, for example the PPARγ/PGC-1α pathway^48, 52^. Further investigations in female offspring are required to confirm this hypothesis.

In the study, the renal mRNA expression of TGFβ1, Col3A and FN in the MHF offspring was unchanged, while TGFβR1 and Col1A mRNA expression was moderately decreased. On the other hand, fibronectin and Col4 protein level was significantly upregulated in MHF offspring, suggesting that maternal HFD consumption can induce renal fibrogenesis in the offspring at weaning. Such effect has been shown to extend to adulthood^9^. Interestingly, only in the male offspring was the Col4 protein expression significantly upregulated. This result further supports higher levels of kidney disorders and damage in male offspring than the female offspring caused by maternal HFD consumption, which is likely linked to increased renal lipid distribution and reduced SIRT1 signalling in the male kidney. It has been demonstrated that SIRT1 activation can suppress renal fibrogenic signalling *in vitro* and *in vivo* ^53^. Whether such therapy is applicable in the offspring at early age to attenuate or reverse the programming effects of maternal HFD consumption on kidney structure requires further investigations.

In summary, the current study demonstrates that maternal HFD consumption can increase renal fat deposition in male offspring and impair renal autophagy and antioxidant defences. Importantly, there are sex-specific differences in the magnitude of effects in the regulatory pathways. In male offspring there is downregulation of the SIRT1/AMPK/FOXO3a/PGC-1α regulatory network; whereas in females, downregulation of PPARγ/PGC-1α is evident. The more marked abnormalities in the male offspring may underpin the well documented increased risk of CKD in males than females in adulthood^23^. Such significant sex differences emphasize the need to independently study both sexes and to employ sex-specific approaches to overcome the adverse effects of maternal obesity on the development of CKD in the offspring. Further investigations with a longitudinal observation targeting SIRT1 signalling pathway are required to confirm sex specificity and the implication of SIRT1 regulation in the development of CKD in the context of maternal obesity.

## Methods

### Animals

The study was approved by the Animal Care and Ethics Committee of the University of Technology Sydney (ACEC# 2009–350). All methods were performed in accordance with the relevant guidelines and regulations in the Australian Code of Practice for the Care and Use of Animals for Scientific Purposes. Female Sprague-Dawley rats (8 weeks) were fed HFD (HFD, 20 kJ/g, 43.5% calorie as fat, Specialty Feed, WA, Australia) or standard rodent chow (11 kJ/g, 14% calorie as fat, Gordon’s Speciality Stockfeeds, NSW, Australia) for 6 weeks before mating, throughout gestation and lactation^54^. On postnatal day (P) 1 the litter size was adjusted to 10 pups/litter (sex ratio controlled at approximately 1:1). Both male and female offspring were investigated. At weaning (P20), all pups were sacrificed after overnight fasting. Blood was collected via cardiac puncture after anaesthesia (Pentothal, 0.1mg/g, i.p., Abbott Australasia Pty Ltd, NSW, Australia). Fat tissues and liver were weighed. The whole kidney was dissected, snap frozen and stored at −80 °C for later analysis. For each type of analysis, 5–6 renal tissues/group were randomly selected.

### Kidney histology

Kidneys were fixed in 10% formalin for 36-h and embedded in paraffin. Paraffin sections were prepared at 4 μm thickness and mount on microscope slides. The sections were stained with hematoxylin and eosin (H&E) for general renal structure and perirenal fat visualisation. Immunohistochemistry (IHC) staining was performed as previously described^55^. Briefly, the tissue sections underwent dewaxing (with xylene), rehydration (with ethanol), antigen-retrieval (99 °C for 20 min in 0.01M, pH 6.0 citric buffer), washing (with water), endogenous peroxidase deactivation with 3% H_2_O_2_ (Sigma-Aldrich, Dublin, Ireland), blocking (with Protein Block Serum-Free, Dako, Glostrup, Denmark), incubation with primary antibodies against fibronectin (1:1000, Abcam, Cambridge, UK) and collagen IV (1:1000, Abcam, Cambridge, UK) followed by biotinylated secondary anti-rabbit IgG antibodies and finally horseradish peroxidase (HRP)-conjugated streptavidin (Dako, Glostrup, Denmark). The tissue specimens were examined by brightfield microscopy (Olympus, Japan). Six consecutive non-overlapping fields from each section of renal cortex were photographed under high magnification. Image J (National Institutes of Health, USA) was used for estimation of the specific staining area. IHC score was determined by log transformation of the staining area.

### Renal protein and lipid extraction

The kidney was homogenized in Triton X-100 lysis buffer (pH 7.4, 150 mM NaOH, 50 mM Tris-HCl, 1% Triton X-100, Roche protease inhibitor) using TissueRuptor (Qiagen, Hilden, Germany). A part of the homogenate was used for lipid extraction according to a published protocol^56^. The lipid extract of each sample was then added to a 96-well plate, followed by incubation with Roche triglyceride reagent GPO-PAP (Roche Life Science, NSW, Australia) at 37 °C for 20 min. The absorbance was read at 490 nm. Glycerine was used in constructing the standard curve.

The remaining tissue homogenate was centrifuged at 10,000 g for 15 min. The supernatant containing total protein was collected and quantitated for protein concentration using Pierce BCA Protein Assay Kit (Thermo Scientific, VIC, Australia) according to the manufacturer’s instructions. The protein concentration of all samples was standardised as 5 μg/μl. The samples were stored at −80 °C for further analyses.

### Quantitative real time PCR (qRT-PCR)

Total RNA was isolated from the kidney tissue using RNeasy Plus Mini Kit (Qiagen Pty Ltd, CA, USA) according to the manufacturer’s instructions. The purified total RNA was used as a template to generate first-strand cDNA using the First Strand cDNA Synthesis Kit (Roche Life Science, NSW, Australia). The amplicons of target genes were amplified with SYBR Green probes. Primer sequences were: SIRT1 (Forward: GCA GGT TGC GGG AAT CCA A, Reverse: GGC AAG ATG CTG TTG CAA A); PPARγ (Forward: 5′-ATC TAC ACG ATG CTG GC-3′, Reverse: 5′-GGA TGT CCT CGA TGG G-3′); PPARα (F: 5′-AGA CAC CCT CTC TCC AGC TTC-3′, R: 5′-GAA TCT TGC AGC TTC GAT CAC-3′); PGC-1α (Forward: AAA CTT GCT AGC GGT CCT CA, Reverse: TGG CTG GTG CCA GTA AGA G-3′); Fatty acid synthase (FAS, Forward: 5′-TGC TCC CAG CTG CAG GC-3′, Reverse: 5′-GCC CGG TAG CTC TGG GTG TA-3′); Sterol regulatory element-binding protein (SREBP)−1c (Forward: 5′-GGA GCC ATG GAT TGC ACA TT-3′, Reverse: 5′-GCT TCC AGA GAG GAG GCC AG-3′), Transforming growth factor beta (TGFβ1, Forward: 5′-TCA GAC ATT CGG GAA GCA GT-3′, Reverse: 5′-ACG CCA GGA ATT GTT GCT AT-3′), TGFβ receptor 1 (TGFβR1, Forward: 5′-CAG CTC CTC ATC GTG TTG G-3′, Reverse: CAG AGG TGG CAG AAA CAC TG-3′), Collagen (Col)1A (Forward: 5′-CAT GTT CAG CTT TGT GGA CCT-3′, Reverse: 5′-GCA GCT GAC TTC AGG GAT GT-3′), Col3A (Forward: 5′-TCC CCT GGA ATC TGT GAA TC-3′, Reverse: 5′- TGA GTC GAA TTG GGG AGA AT-3′), Fibronectin (FN, Forward: 5′-CGG AGA GAG TGC CCC TAC TA-3′, Reverse: 5′-CGA TAT TGG TGA ATC GCA GA-3′) (). Gene expression was standardized to β-actin mRNA (Forward: 5′-ATC GTG CGT GAC ATT AAG; Reverse: 5′-ATT GCC AAT GGT GAT GAC). A sample of the MC-male group was assigned as a calibrator against which all other samples were expressed as fold difference.

### Western-Blot

The proteins were electrophoresed and electroblotted onto the Hybond nitrocellulose membrane (Amersham Pharmacia Biotech, Amersham, UK), which was then incubated with a primary antibody at 4 °C overnight. Antibodies against 5′ AMP-activated protein kinase alpha AMPKα (1:1000), phosphorylated AMPKα (pAMPKα, 1:1000), Autophagy related gene (Atg)6/Beclin-1 (1:1000), Atg12-Atg5 complex (1:2000), Atg7 (1:2000), Atg8/LC3 (1:2000) were obtained from Cell Signalling (MA, USA). FOXO3a (1:2000 LSBio, WA, USA). SIRT1 and Peroxisome proliferator-activated receptor gamma (PPARγ) antibodies were obtained from Santacruz (1:2000, CA, USA). PPARγ coactivator-1 alpha (PGC-1α, 1:2000) and p62 (1:2000) were obtained from Novus Biologicals (VIC, Australia). The anti-oxidative markers manganese superoxide dismutase (MnSOD, 1:2000, Merck Millipore, MA, USA) and Glutathione Peroxidase 1 (GPx-1, 1:200, R&D System, MN, USA) were examined. β-actin (1:2000, Santacruz, CA, USA) was used as the housekeeping protein. All primary antibodies were derived from rabbit except GPx-1 and β-actin antibodies, which were goat-derived. Subsequently the membrane was incubated with a horseradish peroxidase-conjugated secondary antibody (donkey anti-goat for β-actin, otherwise goat anti-rabbit). The immunoblots were developed by adding the Luminata Western HRP Substrates (Millipore, MA, USA) to the membrane and exposed for an appropriate duration using ImageQuant LAS 4000 (Fujifilm, Tokyo, Japan). ImageJ (National Institutes of Health, USA) was used for densitometric analyses.

### Statistical analysis

The results are expressed as mean ± SD and analysed by two-way ANOVA with Bonferroni post hoc test. P values < 0.05 were considered significant.
